# Periostin defines a pathological fibroblast program enriched in restrictive allograft syndrome

**DOI:** 10.1172/jci.insight.203805

**Published:** 2026-08-10

**Authors:** Yudai Miyashita, Taisuke Kaiho, Yuriko Yagi, Taichi Nagano, Xin Wu, Yuanqing Yan, Haiying Sun, Carl Atkinson, GR Scott Budinger, Ankit Bharat, Chitaru Kurihara

**Affiliations:** 1 Division of Thoracic Surgery and; 2 Division of Pulmonary and Critical Care Medicine, Northwestern University Feinberg School of Medicine, Chicago, Illinois, USA.

**Keywords:** Immunology, Pulmonology, Organ transplantation

## Abstract

This study identifies POSTN-positive pathological fibroblasts as key contributors to fibrotic restrictive chronic lung allograft dysfunction after lung transplantation.

**To the Editor:** Chronic lung allograft dysfunction (CLAD) remains a leading cause of late morbidity and mortality after lung transplantation (1). Previously, we reported that fibroblast activation protein (FAP) is selectively induced in activated/pathological fibroblast subsets in CLAD and that quantitative FAP immunostaining in transbronchial biopsies can serve as an early tissue biomarker associated with subsequent CLAD development (2). However, clinical consensus criteria recognize bronchiolitis obliterans syndrome (BOS) and restrictive allograft syndrome (RAS) as distinct CLAD phenotypes with different physiology and histopathology (1), yet the stromal programs that distinguish BOS from RAS in human allograft tissue remain incompletely defined (3).

To address this, we paired single-cell RNA sequencing (scRNA-seq) with matched histopathology from explanted lungs obtained at redo lung transplantation. We analyzed BOS (8 patients; 16 lobar samples) and RAS (3 patients; 15 lobar samples), with phenotype assignment based on longitudinal pulmonary function testing, chest CT findings, and chart review (Figure 1A and Supplemental Tables 1 and 2; supplemental material available online with this article; https://doi.org/10.1172/jci.insight.203805DS1). Integrated scRNA-seq identified major cell types and enabled fibroblast-focused analyses (Supplemental Figure 1, A–D).

Within the stromal compartment, reclustering of fibroblasts identified 4 transcriptional states consistent with previously described lung fibroblast subsets (Figure 1B and Supplemental Figure 1E) (4). Fibroblast-state composition differed by CLAD phenotype at the sample level, with RAS specimens demonstrating a relative expansion of the pathological fibroblast program compared with BOS (Figure 1C). This shift was consistent across lobar sampling within cases (Figure 1D). Fibroblasts in RAS showed selective, but not uniform, changes in fibroblast state marker expression, with increased SERPINE1 and TNC and decreased DCN and INMT (Supplemental Figure 1F).

We next sought fibroblast-derived mediators that best differentiated RAS from BOS. Differential expression analysis comparing RAS and BOS fibroblasts highlighted POSTN among the most strongly RAS-associated transcripts (Figure 1E). POSTN expression was higher in RAS fibroblasts at both single-cell and sample levels (Figure 1, F and G). Across all annotated cell populations, POSTN and COL6A3 were enriched predominantly in fibroblasts, with POSTN-positive cells mapping mainly to the fibroblast compartment on UMAP in both BOS and RAS; within fibroblasts, POSTN expression localized predominantly to the pathological fibroblast state (Supplemental Figure 1, G–J). Mesenchymal subpopulations were summarized into 4 previously reported fibroblast states, with an alternative framework shown in Supplemental Figure 2, A–E. In pathological fibroblasts, POSTN-high cells showed collagen-centric and profibrotic signatures relative to POSTN-low cells (Supplemental Figure 2, F–G). Exploratory AT2 reclustering identified 2 rare subpopulations with increased POSTN expression, but further characterization was beyond the scope of this study (Supplemental Figure 2, H–J).

To determine whether these transcriptomic findings were reflected at the tissue level, we assessed matched histopathology (H&E and Masson’s trichrome) and POSTN immunostaining (Figure 1H and Supplemental Figure 2K). Compared with BOS, RAS explants demonstrated more extensive fibrotic remodeling by Masson’s trichrome staining and increased POSTN immunoreactivity. Quantitative scoring showed higher periairway and parenchymal fibrosis in RAS than in BOS, with a higher POSTN-positive area fraction; other compartments showed no clear phenotype-associated differences (Figure 1I).

These findings provide a convergent single-cell and tissue-level framework linking RAS to a fibroblast program characterized by pathological-state expansion and increased periostin. Prior work in idiopathic pulmonary fibrosis has described periostin as a fibroblast-associated extracellular matrix mediator and biomarker of fibrotic remodeling (5, 6), supporting the concept that RAS and IPF may share downstream profibrotic pathways. In this context, our data suggest that POSTN upregulation is associated with a pathological fibroblast state enriched in RAS, while increased periostin deposition in explanted allografts may also reflect the greater overall fibrotic burden in RAS.

From a translational perspective, periostin may be informative in two complementary ways. POSTN-high pathological fibroblasts provide a cellular context for the matrix-rich remodeling that typifies RAS, and tissue periostin deposition aligned with parenchymal and periairway fibrosis. Although we did not establish predictive performance, these parallel single-cell and histologic readouts support testing whether periostin quantification can complement pathology-based CLAD phenotyping.

Although FAP can serve as an early CLAD biomarker, the timing and phenotypic specificity of periostin in CLAD are unknown (2). The cohort was modest and restricted to explanted lungs at redo transplantation, capturing end-stage disease. We therefore cannot determine whether periostin elevation precedes clinical RAS, tracks disease trajectory, or reflects established fibrosis. In addition, our primary analytic unit was the lobar specimen, and larger patient-level longitudinal studies will be needed for validation. Finally, while our data localized POSTN to a pathological fibroblast state and showed strong tissue-level concordance, they did not determine whether periostin is mechanistically required for restrictive remodeling in CLAD.

In summary, our results support an association between RAS and increased POSTN signal at the single-cell and tissue levels; however, these findings may reflect both enrichment of a POSTN-high pathological fibroblast program and the greater overall fibrotic burden in RAS explants. These observations motivate future multicenter studies that integrate longitudinal sampling, standardized phenotyping, and orthogonal assays to determine whether periostin can serve as a robust marker of restrictive CLAD biology and whether targeting periostin-positive fibroblast extracellular matrix programs can be leveraged therapeutically.

For detailed methods, information regarding sex as a biological variable, statistics, study approval, author contributions, and acknowledgments, see the supplemental materials.

The sequencing data are available in the Gene Expression Omnibus (GSE325801). Values underlying graphed data and reported means are in the Supporting Data Values file. Additional details are in the Supplemental Methods.

## Funding support

This work is the result of NIH funding, in whole or in part, and is subject to the NIH Public Access Policy. Through acceptance of this federal funding, the NIH has been given a right to make the work publicly available in PubMed Central.

- NIH grant R01HL176632 to CK.

## Conflict of interest

The authors have declared that no conflict of interest exists.

## Supplementary Material

Supplemental data

Supporting data values

## Figures and Tables

**Figure 1:**
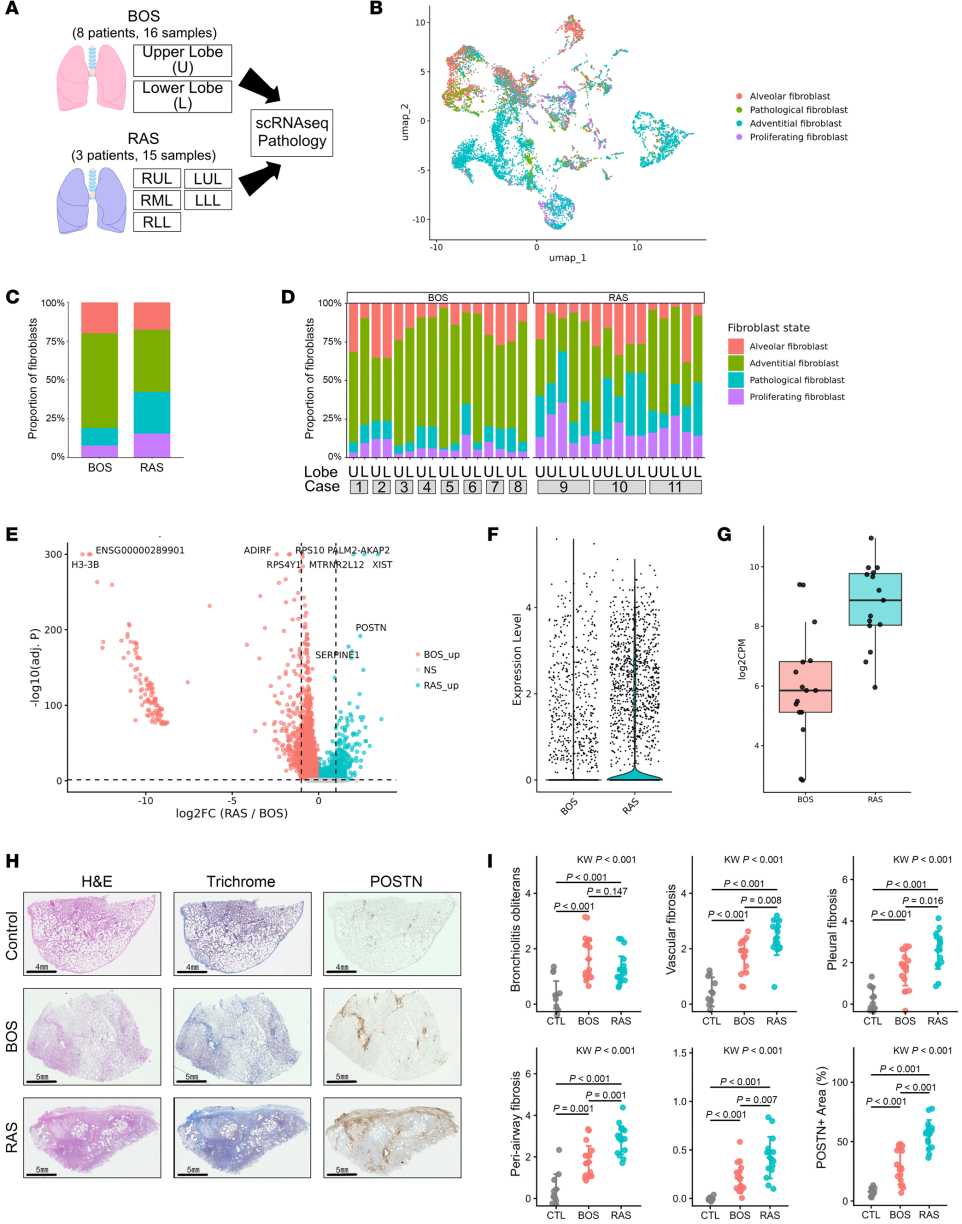
POSTN marks a pathological fibroblast program enriched in RAS. (**A**) Study design. (**B**) UMAP of 4 fibroblast states. (**C** and **D**) Fibroblast state proportions by phenotype and individual lobar samples. (**E**) Differential expression in fibroblasts (RAS vs. BOS). (**F**) Single-cell POSTN expression in fibroblasts. (**G**) Sample-level POSTN abundance. (**H**) Representative H&E, trichrome, and POSTN immunostaining. (**I**) Histologic scoring and POSTN-positive area fraction. The Kruskal-Wallis test followed by Dunn’s multiple-comparison test was used. Box-and-whisker plots show the median (center line), the interquartile range (box), and whiskers extending to 1.5× the interquartile range; individual points represent lobar specimens.

## Version 1 08/10/2026

Electronic publication
