# Supplementary material for: Periostin defines a pathological fibroblast program enriched in restrictive allograft syndrome
Source: JCI Insight. 2026 Aug 10;11(15):e203805. doi: 10.1172/jci.insight.203805 (PMC13463613; doi:10.1172/jci.insight.203805)
Supplement: Supplementary file 1 [file jciinsight-11-203805-s093.pdf]

Supplemental information for

Periostin Defines a Pathological Fibroblast Program Enriched in Restrictive Allograft Syndrome

Yudai Miyashita, Taisuke Kaiho, Yuriko Yagi, Taichi Nagano, Xin Wu, Yuanqing Yan, Haiying Sun, Carl Atkinson, GR Scott Budinger, Ankit Bharat, Chitaru Kurihara

Corresponding Author:

Chitaru Kurihara, MD

E-mail; chitaru.kurihara@northwestern.edu

This supplemental file includes:

Supplemental Figure S1-2

Supplemental Table S1-2

Supplemental Methods

Data availability

Author contributions

Acknowledgments

Supplemental Figure S1

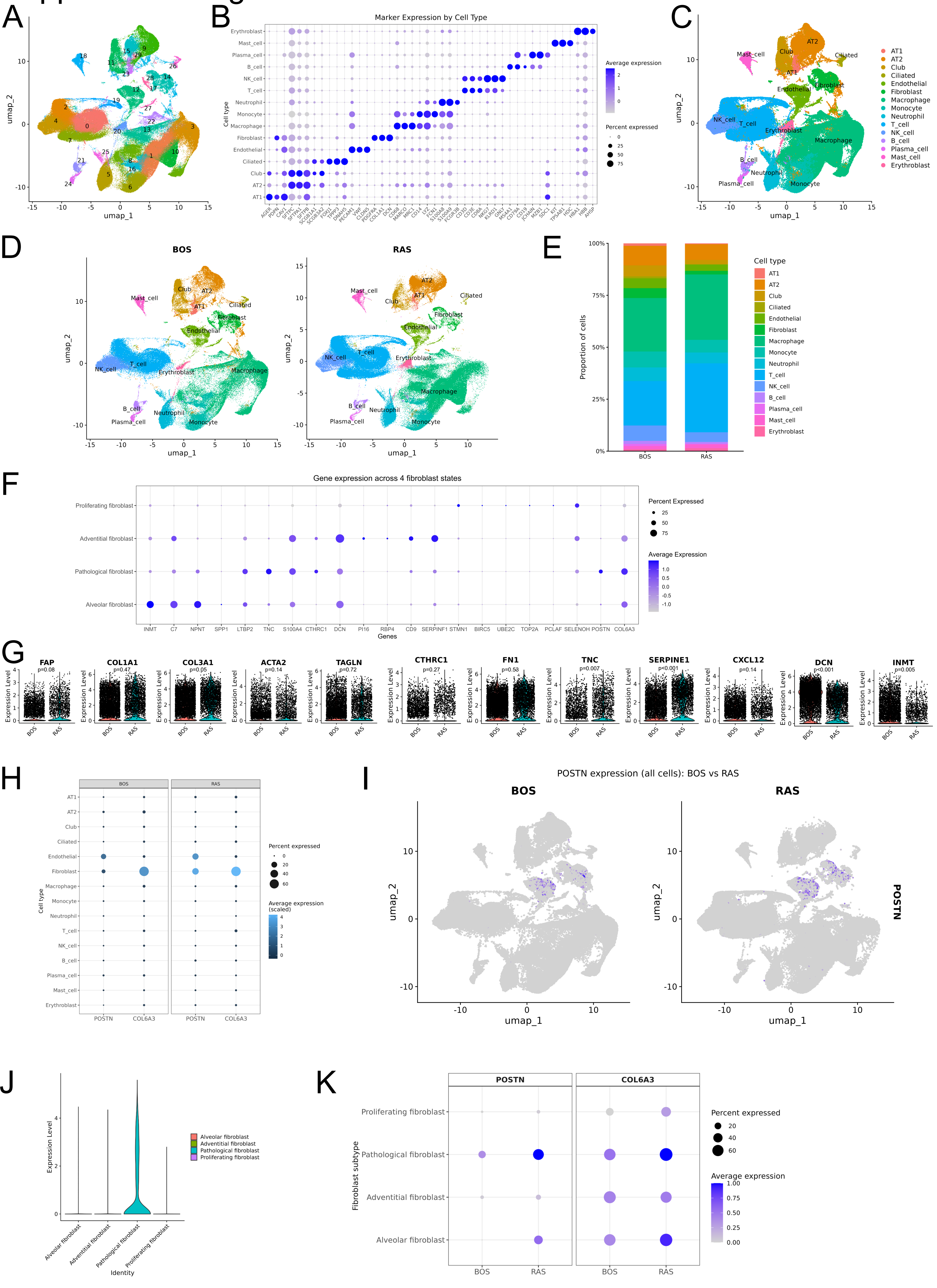

Supplemental Figure S1. Integrated single-cell atlas identifies fibroblasts as the major source of POSTN and COL6A3 in RAS lungs.

(A) UMAP of all cells showing unsupervised clusters. (B) Dot plot of canonical marker genes used to assign major cell types; dot size indicates the fraction of expressing cells and color indicates scaled average expression. (C) UMAP colored by annotated cell type. (D) UMAPs split by CLAD phenotype (BOS and RAS) showing the distribution of major cell populations. (E) Stacked bar plot showing the relative proportions of annotated cell types in BOS and RAS. (F) Dot plot showing expression of selected marker genes across the four fibroblast states: alveolar, adventitial, pathological, and proliferating fibroblasts. (G) Violin plots comparing expression of representative fibroblast- and matrix-associated genes in BOS versus RAS fibroblasts. (H) Dot plot showing POSTN and COL6A3 expression across all annotated cell types in BOS and RAS. (I) Feature plots showing POSTN expression across all cells in BOS and RAS. (J) Violin plot showing POSTN expression across the four fibroblast states. (K) Dot plot comparing POSTN and COL6A3 expression across fibroblast states in BOS and RAS.

Supplemental Figure S2

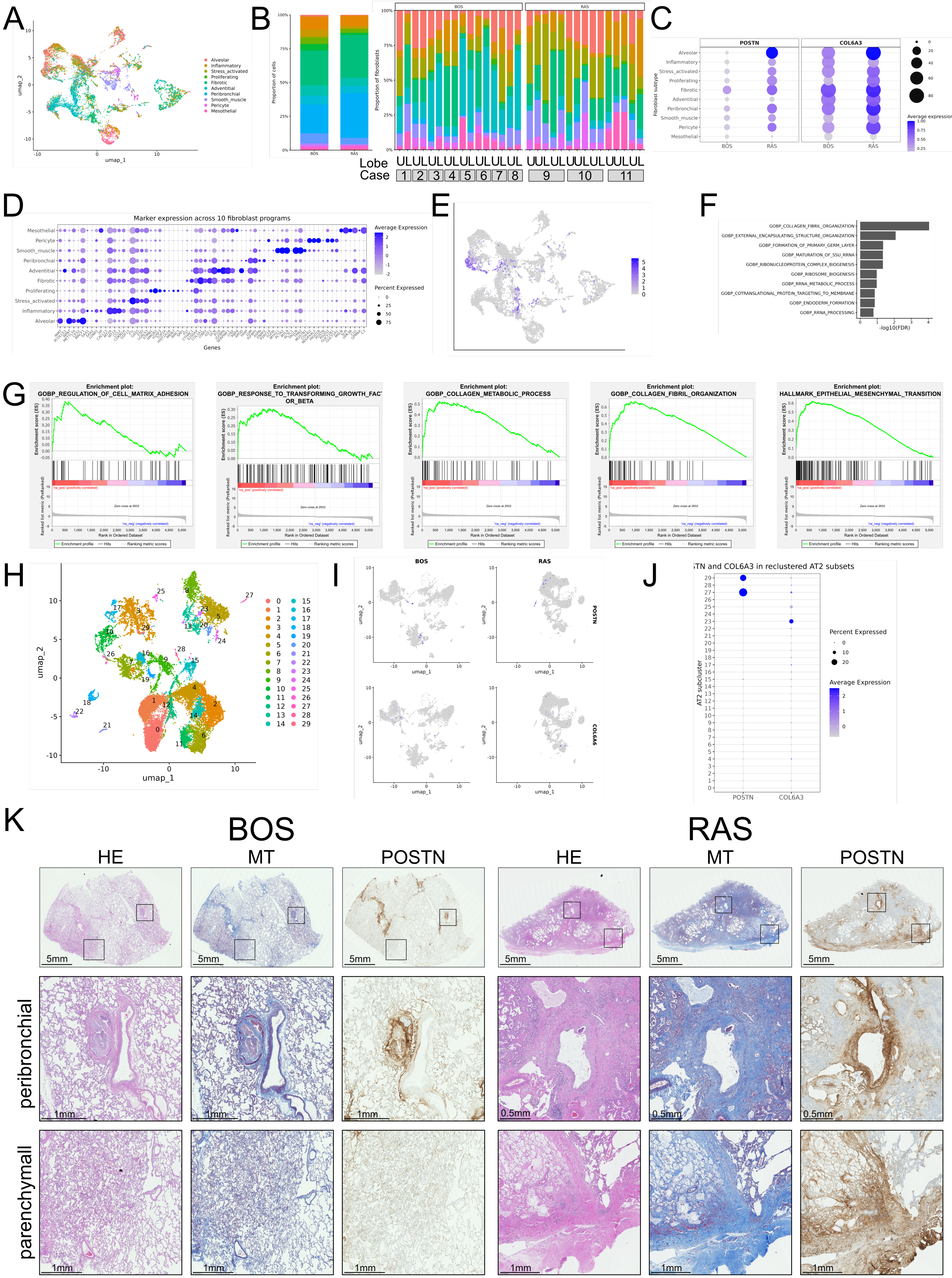

Supplemental Figure S2. Higher-resolution fibroblast and AT2 analyses further define RAS-associated POSTN-high fibrotic programs and their histologic localization.

(A) UMAP of fibroblasts reclustered into 10 fibroblast programs/states. (B) Stacked bar plots showing the relative abundance of fibroblast programs overall in BOS and RAS (left) and by individual case and sampled lobe (right). (C) Dot plot comparing POSTN and COL6A3 expression across fibroblast programs in BOS and RAS. (D) Dot plot of representative marker genes across the 10 fibroblast programs. (E) Feature plot showing expression of the representative fibrosis-associated gene/program on the fibroblast UMAP. (F) Top Gene Ontology biological process terms enriched in the RAS-associated fibrotic fibroblast program. (G) Gene set enrichment plots showing enrichment of extracellular matrix adhesion, response to transforming growth factor-β, collagen metabolic process, collagen fibril organization, and epithelial–mesenchymal transition pathways. (H) Higher-resolution subclustering of AT2 cells. (I) Feature plots showing POSTN and COL6A6 expression in AT2 subclusters in BOS and RAS. (J) Dot plot of POSTN and COL6A3 expression across AT2 subclusters. (K) Representative whole-section and higher-magnification images of H&E, Masson's trichrome (MT), and POSTN immunostaining in BOS and RAS lungs, highlighting peribronchial and parenchymal regions. Scale bars are shown in each panel.

Abbreviations: UMAP, Uniform Manifold Approximation and Projection; scRNA-seq, single-cell RNA sequencing; CLAD, chronic lung allograft dysfunction; BOS, bronchiolitis obliterans syndrome; RAS, restrictive allograft syndrome; ECM, extracellular matrix; FAP, fibroblast activation protein; COL1A1, collagen type I alpha 1 chain; COL3A1, collagen type III alpha 1 chain; ACTA2, actin alpha 2 (smooth muscle); TAGLN, transgelin; CTHRC1, collagen triple helix repeat containing 1; FN1, fibronectin 1; TNC, tenascin C; SERPINE1, serpin family E member 1 (PAI-1); CXCL12, C-X-C motif chemokine ligand 12; POSTN, periostin; GO, Gene Ontology; BP, Biological Process; GSEA, Gene Set Enrichment Analysis; TGF-β, transforming growth factor beta.

Supplemental Table S1. Patient Characteristics

Case	CLAD phenotype	Age (years)	Ethnicity	Sex	BMI (kg/m^2)	Indication for primary LTx	Time from primary to redo LTx (Months)	Primary LTx type	Redo LTx type
1	BOS	55	White	Male	22.6	ILD	15	Single	Bilateral
2	BOS	36	Hispanic	Female	16.0	Sarcoidosis	13	Bilateral	Bilateral
3	BOS	64	White	Male	27.1	COPD	83	Bilateral	Bilateral
4	BOS	30	Other	Male	21.3	Cystic fibrosis	28	Bilateral	Bilateral
5	BOS	61	White	Male	24.1	ARDS	42	Bilateral	Bilateral
6	BOS	43	White	Female	25.3	Cystic fibrosis	59	Bilateral	Bilateral
7	BOS	56	Other	Male	21.1	Chronic HP	52	Bilateral	Bilateral
8	RAS	17	Hispanic	Male	29.2	PVOD	36	Bilateral	Bilateral
9	RAS	27	Other	Female	17.9	Cystic fibrosis	56	Bilateral	Bilateral

Data are presented at the patient level. Age and BMI are reported at the time of redo lung transplantation. Ethnicity was self-reported. Abbreviations: ARDS, acute respiratory distress syndrome; BMI, body mass index; BOS, bronchiolitis obliterans syndrome; CLAD, chronic lung allograft dysfunction; COPD, chronic obstructive pulmonary disease; HP, hypersensitivity pneumonitis; ILD, interstitial lung disease; LTx, lung transplantation; PVOD, pulmonary veno-occlusive disease; RAS, restrictive allograft syndrome.

Supplemental Table S2. Clinical features supporting BOS versus RAS phenotype assignment

Case	CLAD phenotype	FEV1 decline from post-transplant baseline (%)	TLC decline from baseline (%)	Key pulmonary function pattern	Chest CT findings supporting phenotype	Clinical trajectory of CLAD	Time from CLAD onset to redo LTx (months)	Basis for phenotype assignment
1	BOS	56	NA	Predominantly obstructive	Air trapping / no persistent parenchymal opacity	Progressive obstructive decline	10	PFT + CT + chart review
2	BOS	78	NA	Predominantly obstructive	mosaic attenuation / no persistent parenchymal opacity	Progressive obstructive decline	3	PFT + CT + chart review
3	BOS	48	NA	Predominantly obstructive	Air trapping / no persistent parenchymal opacity	Progressive obstructive decline	2	PFT + CT + chart review
4	BOS	80	NA	Predominantly obstructive	Air trapping / mosaic attenuation / no persistent parenchymal opacity	Progressive obstructive decline	6	PFT + CT + chart review
5	BOS	70	NA	Predominantly obstructive	Air trapping / no persistent parenchymal opacity	Progressive obstructive decline	2	PFT + CT + chart review
6	BOS	77	NA	Predominantly obstructive	No persistent parenchymal opacity	Progressive obstructive decline	1	PFT + CT + chart review
7	BOS	67	NA	Predominantly obstructive	Air trapping / mosaic attenuation / no persistent parenchymal opacity	Progressive obstructive decline	10	PFT + CT + chart review
8	BOS	68	NA	Predominantly obstructive	Air trapping / mosaic attenuation / no persistent parenchymal opacity	Progressive obstructive decline	8	PFT + CT + chart review
9	RAS	69	NA	Restrictive	Persistent parenchymal opacity / pleuroparenchymal fibrosis / volume loss	Restrictive decline with radiographic fibrosis	5	PFT + CT + chart review
10	RAS	66	12	Restrictive	Persistent parenchymal opacity / pleuroparenchymal fibrosis / volume loss	Restrictive decline with radiographic fibrosis	5	PFT + CT + chart review
11	RAS	31	NA	Restrictive	Persistent parenchymal opacity / pleuroparenchymal fibrosis / volume loss	Restrictive decline with radiographic fibrosis	1	PFT + CT + chart review

Data are presented at the patient level. Pulmonary function decline was assessed relative to each patient's post-transplant baseline. CLAD phenotype assignment was based on longitudinal pulmonary function testing, chest CT findings, and chart review. TLC measurements were not available in all cases. Phenotype assignment was based on longitudinal spirometry, chest CT findings, and chart review. Because total lung capacity measurements were not routinely available, restrictive physiology was adjudicated using the overall spirometric pattern together with persistent radiographic abnormalities.

Supplemental Methods

Sex as a biological variable

This study analyzed explanted lung allograft tissue obtained at the time of redo lung transplantation. Recipient sex was recorded from the medical record and was not used to determine eligibility or sampling. The cohort included 6 male and 3 female recipients (BOS: 7, RAS: 2). Because of the modest cohort size and the limited number of RAS cases, the study was not powered to perform sex-stratified analyses or to test sex-by-phenotype interactions. Accordingly, all primary analyses were performed across sexes. The biological processes evaluated (fibroblast-state composition, POSTN expression, and histologic fibrosis/periostin deposition) are expected to be relevant across sexes; however, larger cohorts will be required to determine whether the magnitude of these associations differs by sex.

Human Lung Tissues and patient characteristics

All the lung specimens were obtained from lung transplantation surgery. The diagnosis and classification of CLAD was made by a consensus report from the Pulmonary Council of the ISHLT [1]. It is defined as a sustained (at least 3 months) decline in the forced expiratory volume in one second (FEV_1) of at least 20% from the post-transplant baseline, in the absence of other reversible causes. CLAD is further sub-classified into phenotypes such as bronchiolitis obliterans syndrome (BOS) and restrictive allograft syndrome (RAS) based on clinical, radiologic, and physiologic criteria [1].

Single-cell RNA-seq

Single-cell RNA-seq analyses were performed on human lung allograft explant samples obtained at redo lung transplantation. Raw base call files were demultiplexed into FASTQ files using Cell Ranger mkfastq (v8.0.1, 10x Genomics). Gene expression count matrices were generated using Cell Ranger count (v8.0.1) with the 10x Genomics GRCh38-2024-A reference. Downstream analyses were conducted in Seurat (v4.0) in R. Cells were filtered based on standard quality-control metrics to remove low-quality droplets, and data were log-

normalized followed by variable feature selection. Samples were integrated using Seurat's anchor-based workflow (FindIntegrationAnchors/IntegrateData), followed by PCA and UMAP for dimensional reduction. Graph-based clustering was performed using a resolution of 0.5, and clusters were annotated using canonical marker genes reported in prior studies. For indicated contrasts, we subsetted the fibroblast compartment and performed differential expression using Seurat FindMarkers with the Wilcoxon rank-sum test on normalized expression values from the RNA assay. Unless otherwise noted, genes meeting $|\log2 \text{ fold change}| \geq 0.25$ and Benjamini–Hochberg FDR-adjusted $P < 0.05$ were considered differentially expressed. Differentially expressed gene lists were subjected to over-representation analysis using g:Profiler (g:GOSt; organism = hsapiens). The background gene universe was restricted to genes detected in the corresponding fibroblast dataset. Multiple-testing correction used g:Profiler's default g:SCS procedure, and terms with adjusted $P < 0.05$ were considered significant.

Histology and Immunostaining

Hematoxylin and eosin staining and Masson's trichrome staining were performed using standard protocols for the analysis of fibrosis scoring. Standard immunohistochemistry analysis was performed using Periostin antibody (Abcam ab215199) diluted at 1:750. The immunohistochemistry staining was performed on the Leica Bond-Max. Slides were baked before being deparaffinized. Heated epitope retrieval was performed with Epitope Retrieval Solution 2 (pH 9) (Leica AR9640) for 20 minutes. Endogenous peroxidase was neutralized with a peroxidase block for 5 minutes, before primary was added to the slides and incubated for 15 minutes. Post primary incubation, the polymer was applied to the slide and incubated for 8 minutes. Finally, the reaction products were visualized with DAB and counterstained with hematoxylin. Slides were then dehydrated and mounted with xylene-based mounting media. Full-specimen, stitched images at ×40 magnification were captured using a Nanozoomer 2.0 HT (Hamamatsu, Shizuoka, Japan).

Histopathology scoring.

Formalin-fixed, paraffin-embedded sections were prepared from explanted lung allograft tissue obtained at redo lung transplantation. Serial sections were stained with hematoxylin and eosin (H&E) and Masson's trichrome for assessment of airway-centered and parenchymal remodeling[2]. To enable standardized comparison

across lobar specimens, we applied a semi-quantitative histopathology scoring framework adapted from a published CLAD explant pathology template [3]. Each lobar specimen was scored for the following features: peri-airway fibrosis, bronchiolitis obliterans, parenchymal fibrosis, pleural fibrosis, and vascular fibrosis. Features were graded on an ordinal scale (e.g., 0 = absent, 1 = mild, 2 = moderate, 3 = severe). Values were analyzed at the lobar-sample level.

Computer-based Artificial Intelligence Analysis of Immunohistochemical Staining Images

DAB-stained slides were fully scanned with a Nanozoomer 2.0 HT at ×40 objective magnification to obtain the whole slide image (WSI). An open-source tool called Orbit Image Analysis (www.orbit.bio) was used for further analysis of the IHC staining signals [2,4,5]. Briefly, the IHC analysis model was trained on the basis of a sliding window feature extraction approach, which uses different features as predictors to classify the center pixel label to discriminate regions within the image, such as normal lung alveoli and airways, hematoxylin-stained nucleus, DAB-stained positive signals, and blank parts outside the tissue and within the airways. A training set of representative DAB-stained images were used to train the machine learning model. The IHC analysis model can sensitively recognize the different components within the slides. For measurement of the percentage of Periostin positive areas, each tissue slice was divided into three parts (Periostin positive, Periostin negative, and blank). Then the ratio of the Periostin positive area to the total area without blank was calculated. To assess reproducibility, the AI analysis model used for quantifying Periostin-positive areas was saved and can be consistently reapplied. When the same specimens were re-analyzed using the saved model parameters, identical results were obtained, confirming the model's internal consistency and reproducibility for the same input. All WSIs from both institutions were processed together in a single batch with the same trained model, and the entire analysis was conducted by the same operator to ensure consistency across sites.

Statistical analysis

All statistical analyses were performed in R (version 4.5.1). Unless otherwise specified, tests were two-sided and a P value < 0.05 was considered statistically significant. The primary analytic unit was the lobar specimen or per-cell expression distributions (sample-level analysis). For comparisons between BOS and RAS at the sample level (e.g., sample-level POSTN abundance and histologic fibrosis/periostin quantification), we used

Welch's two-sample t test to allow for unequal variances. For single-cell differential expression analyses in fibroblasts (RAS vs BOS), we used Seurat's differential expression framework (FindMarkers) with the Wilcoxon rank-sum test and controlled for multiple testing using the Benjamini–Hochberg false discovery rate (FDR); adjusted P values are reported for genome-wide analyses (e.g., volcano plots). Gene Ontology Biological Process enrichment and gene set enrichment analysis (GSEA) were performed on ranked differential expression statistics, with gene set significance assessed using permutation-based methods and multiple-testing correction by FDR where applicable; enrichment results are reported with adjusted P values. Data are shown as individual lobar samples or individual cells, as indicated in the figure legends.

Study approval

The study on clinical samples was approved by the Ethics Committee of the Northwestern Memorial Hospital (STU00212120). Before collection, the written consents for tissue harvesting were obtained from each patient or family member. Patient data were collected retrospectively using electronic medical records and stored in a database at the Northwestern University Medical Center.

Data availability

The scRNA-seq data generated in this study have been deposited in the NCBI Gene Expression Omnibus under accession number GSE325801. Deidentified individual participant data (including data dictionaries) supporting the findings of this study will be made available upon reasonable request. Researchers seeking access to the data should contact the corresponding author at chitaru.kurihara@northwestern.edu. Access will be granted to investigators whose proposals align with the aims of this research, subject to approval. Additional documents, such as the study protocol and statistical analysis plan, will also be available upon request. Data will be accessible starting from the publication date with no end date.

Author contributions

Y.M. and C.K. conceived and designed the study, performed experiments, interpreted the data, and wrote the manuscript. T.K., Y.Yan..,conducted data analysis. Y.Yagi., and C.K. collected human lung specimens. H.S.

contributed to data interpretation and manuscript writing. A.B., and C.K. interpreted the data, contributed to manuscript writing, and provided funding and resources.

Acknowledgments

This work was supported by the Northwestern University Pathology Core Facility and a Cancer Center Support Grant (NCI CA060553). We thank Elena Susan for English proofreading. This research was supported in part through the computational resources and staff contributions provided by the Genomics Computing Cluster (Genomic Nodes on Quest), which is jointly supported by the Feinberg School of Medicine, the Center for Genetic Medicine, and Feinberg's Department of Biochemistry and Molecular Genetics, the Office of the Provost, the Office for Research, and Northwestern Information Technology. The Genomics Computing Cluster is part of Quest, Northwestern University's high-performance computing facility, with the purpose to advance research in genomics.

Reference

1. Verleden GM, Glanville AR, Lease ED, et al. Chronic lung allograft dysfunction: Definition, diagnostic criteria, and approaches to treatment—A consensus report from the Pulmonary Council of the ISHLT. *Journal of Heart and Lung Transplantation*. 2019;38(5). doi:10.1016/j.healun.2019.03.009

2. Miyashita Y, Kaiho T, Nagata H, et al. Exploring fibroblast activation protein as an early biomarker in chronic lung allograft dysfunction. *European Respiratory Journal*. Published online 2025:2501738. doi:10.1183/13993003.01738-2025

3. Renaud-Picard B, Berra G, Hwang D, et al. Spectrum of chronic lung allograft dysfunction pathology in human lung transplantation. *Journal of Heart and Lung Transplantation*. 2024;43(10):1701-1715. doi:10.1016/j.healun.2024.04.002

4. Yang P, Luo Q, Wang X, et al. Comprehensive Analysis of Fibroblast Activation Protein Expression in Interstitial Lung Diseases. *Am J Respir Crit Care Med*. 2023;207(2):160-172. doi:10.1164/rccm.202110-2414OC

5. Stritt M, Stalder AK, Vezzali E. Orbit image analysis: An open-source whole slide image analysis tool. *PLoS Comput Biol*. 2020;16(2). doi:10.1371/journal.pcbi.1007313
